# Toxic Effects of Lead Exposure on Freshwater Climbing Perch, *Anabas testudineus*, and Bioremediation Using *Ocimum sanctum* Leaf Powder

**DOI:** 10.3390/toxics12120927

**Published:** 2024-12-20

**Authors:** Nimai Chandra Saha, Arnab Chatterjee, Priyajit Banerjee, Ritwick Bhattacharya, Auroshree Sadhu, Paolo Pastorino, Shubhajit Saha

**Affiliations:** 1 Department of Zoology, Bidhannagar College, Bidhannagar, Kolkata 700064, West Bengal, India; 2 Ecotoxicology Research Laboratory, Department of Zoology, The University of Burdwan, Burdwan 713104, West Bengal, India; arnab.chat93@gmail.com (A.C.); ritwick111@gmail.com (R.B.); auroshreesadhu7@gmail.com (A.S.); ssaha@zoo.buruniv.ac.in (S.S.); 3 Department of Biotechnology, Swami Vivekananda University, West Bengal 700121, India; priyajit.research@gmail.com; 4 Istituto Zooprofilattico Sperimentale del Piemonte, Liguria e Valle d’Aosta, 10154 Torino, Italy

**Keywords:** growth, haematological biomarkers, hepatosomatic index, lead toxicity, oxidative stress enzymes

## Abstract

The acute and chronic toxicity of lead to *Anabas testudineus* was determined in this study using static replacement bioassay testing. During the chronic toxicity studies, an experiment on the bioremediation of lead toxicity using *Ocimum sanctum* leaf powder was conducted. The 96 h LC_50_ values of lead for *Anabas testudineus* was 1.08 mg/L. Different biomarkers, such as the hepatosomatic index, gonadosomatic index, and fecundity, were significantly lower in fish subjected to 10% and 20% of the 96 h LC_50_ values of lead, compared to controls. The 45-day chronic exposure of fish to lead concentrations of 0.2 mg/L and above significantly lowered the number of total RBC, hemoglobin content, HCT (%), plasma protein, and cholesterol while decreasing the level of total WBC, plasma glucose, creatinine, serum AST and serum ALT. The leaf powder of *Ocimum sanctum* plays a significant role in ameliorating lead toxicity.

## 1. Introduction

Due to the harmful effects on aquatic species and human health, the toxicity of various chemicals in the environment, particularly in water sources, resulting from both natural and human activities, has become a major concern [1,2,3,4,5]. These chemicals adversely affect the survival, growth, well-being, and behaviour of fish [6,7,8,9,10]. Lead (Pb) has been identified as one of the most important and versatile metal ions used in industry, with many applications in metal finishing, storage batteries, paints, and electroplating [11]. Lead, with atomic number 82, is abundant in the environment, ranking 36th in natural abundance [12]. According to WHO guidelines, lead’s maximum acceptable limit in drinking water is 0.01 mg/L. Natural sources of lead include volcanic eruptions, marls, gypsum, sea aerosol particles, and biological cycling processes, including the weathering of lead-containing rocks and soils [13].

Furthermore, Pb exposure acts as an immune toxicant in fish, affecting their immune responses [13]. Lead pollution poses a severe environmental health threat due to its non-biodegradability and its long-term detrimental effects through accumulation [13]. Excessive intake of Pb ions can result in damage to the nervous system, brain, kidneys, reproductive system, and even death. Lead contamination has garnered increasing global attention due to its toxic effects on fish, humans, and the environment [14]. Pb toxicity has devastating consequences on fish populations, adversely affecting various metabolic processes [14]. It also triggers energy-consuming detoxification mechanisms, reducing the energy available for growth [15]. Moreover, lead acts as an endocrine disruptor in fish. Its pro-oxidative properties can induce oxidative stress in fish, leading to oxidative damage to cell membranes [15]. Reports on Pb toxicity in fish and other aquatic organisms highlight the need for further research to better understand lead poisoning in natural water bodies. Consequently, this study aims to estimate the acute and chronic toxicity of Pb to aquatic organisms, assess Pb concentrations in water, and explore bioremediation strategies for safe lead disposal. The objective of this study is to assess the acute and chronic toxicity of lead on fish (*Anabas testudineus*) using static replacement bioassay tests.

Plants absorb a wide range of substances from the soil, some of which have unknown biological function, while others can be harmful even at low concentrations [16]. Since plants form the base of the food chain, there is concern that harmful elements may be transmitted from plants to higher trophic levels [17]. Utilisation of naturally available aromatic and herbs-medicinal plants in feeding the fish is still less on the experimental and commercial level. However, few studies have been conducted to utilize these herbs and plants as feed additives to enhance growth and feed efficiency and also for bioremediation potential during chronic toxicity experiments to mitigate toxicity of different contaminants [18]. Basil (*Ocimum* spp.) meets the criteria as it adapts well to warm environments, does not reproduce aggressively, and does not spread uncontrollably [19]. In this study, the leaf powder of *Ocimum sanctum* was tested for its bioremediation potential during chronic toxicity experiments to mitigate lead toxicity.

## 2. Material and Methods

### 2.1. Test Organisms

Adult specimens (n = 256) of the freshwater, air-breathing fish, *Anabas testudineus* [weight 38.24 ± 1.9 g (mean ± SD); length 12.3 ± 2.9 cm (mean ± SD)] used in the study were bought from a fish farm at Burdwan, West Bengal. They were then transferred to the Aquatic Toxicology Laboratory, Department of Zoology, The University of Burdwan, West Bengal.

### 2.2. Test Chemical

As the test chemical, analytical grade lead oxide (also called lead monoxide), PbO, with 99.99% purity (molecular weight 300.59 g/mol; Sigma Aldrich Inc., Mumbai, India) was used.

### 2.3. Acute Toxicity Test

#### 2.3.1. Experimental Set Up

Static replacement bioassays were conducted in the laboratory conditions, following the methods described in APHA [20] and Kaviraj et al. [21]. Tube-well water stored beforehand in a tank was used as the diluents medium in the bioassays [22]. The physicochemical water parameters measured prior to treatment were (mean ± SD) as follows: temperature 28.5 ± 1.5 °C, pH 7.11 ± 0.13, CO_2_ 12.57 ± 1.24 mg/L, dissolved oxygen (DO) 5.85 ± 0.92 mg/L, total alkalinity 175.00 ± 8.07 mg/L as CaCO_3,_ and hardness 106.00 ± 6.70 mg/L as CaCO_3_. The test organisms were acclimatized to the test condition for 15 days before starting the bioassay. Only healthy specimens were selected at random from a single stock, regardless of sex, before the experiment [23]. Sufficient control was maintained for all bioassay tests [24]. The entire group of test organisms was discarded if mortality in the control group exceeded 5%. LC_50_ values for the test chemicals, along with changes in fish behaviour and respiratory rates, were evaluated during 96 h acute toxicity tests using various nominal concentrations (0.5, 0.7, 0.9, 1.1, 1.3, 1.5, 1.7, 1.9, 2.1, 2.3 and 2.5 mg/L). The exposure concentrations were chosen using the 24 h range finding test [25,26]. Additionally, a 96-h feeding test was conducted with sublethal concentrations of the test chemicals. For the 45-day chronic toxicity tests, sublethal doses were used to assess changes in fish growth rate, haematologicaland biochemical parameters, serum enzyme levels, and histopathology, as well as alterations in the physicochemical properties of the test water. The test bioassays were conducted according to the regulations approved by the Institutional Biosafety Committee, The University of Burdwan (BU/IBSC/22/Zo/36).

#### 2.3.2. Respiratory Rate Test

The respiratory rate changes of fish exposed to different lethal concentrations of the toxicants were estimated from the opercula movements of fish/minute, following the methods of Kaviraj, Bhunia, and Saha [21], and Chukwuka et al. [27] during their acute exposure. The total number of opercula movements per minute per fish was evaluated. Six such observations (two from each replicate) were recorded at random for each concentration every 24 h. The respiratory test was conducted for 96 h in 15 L glass aquaria, each holding 10 L of water with four fish. Three replicates of such aquaria were exposed to each concentration of the toxicant and control. Opercula movements were counted at 10.00 A.M. and 4.00 P.M. daily. The opercular movements per minute were recorded at random intervals from two individuals in each aquarium during each observation. To facilitate accurate counting, the aquaria were well-illuminated, with a light source positioned behind each aquarium.

#### 2.3.3. Test for Changes in Behaviour

Behavioral changes in fish exposed to various lethal concentrations of Pb were observed visually during the 96-h acute exposure, following standard protocols [26,27,28,29]. Behavioural tests for *Anabas testudineus* were performed in 15 L glass aquaria, each containing 10 L of water. Three replicates of aquaria were used for each toxicant concentration.

#### 2.3.4. Feeding Test

The feeding studies were conducted in 15 L glass aquaria holding 10 L of water, with three adult fish per tank, over a 96-h period using static renewal bioassays. Each test chemical was evaluated at three sublethal concentrations: 10%, 20%, and a combination of 20% of the 96 h LC_50_ value with *Ocimum sanctum* leaf powder, along with a control. The leaf powder comprised one-quarter of the fish’s daily meal. The basil powder was mixed with white fish meal, wheat flour, shrimp meal, dried yeast, and soybean meal. The sublethal concentrations were determined based on the 96 h LC_50_ values of the toxicants. Three aquaria were assigned to each sublethal concentration and the control, following a randomized block design. The fish were fed freshly chopped earthworms for four hours daily, starting at 8 am. Each day, the water was drained and replaced with fresh water containing the appropriate test chemical. Unconsumed food was measured and removed to prevent organic decomposition. The food consumed (in %) was calculated as difference between the total wet weight of food provided and the food left unconsumed. The control fish consumed 100% of the food. Fish food consumption at each sublethal test concentration was compared to the control.

### 2.4. Chronic Toxicity Test

#### 2.4.1. Experimental Set Up

Chronic toxicity tests were conducted over a 45-day period during the monsoon season in the laboratory, using 15 L glass aquaria containing 10 L of dechlorinated tap water. For the preparation of the *Ocimum sanctum* leaf powder, the *O. sanctum* leaves were collected from a nearby source and cleaned thoroughly with distilled water. The leaves were then dried in an oven at 45 °C for 48 h and ground to powder using the mortar and pestle. Based on the results of the acute toxicity tests (96 h LC_50_ values), three sublethal concentrations of each test chemical were determined: 10% of the 96 h LC_50_ value, 20% of the 96 h LC_50_ value, and a mixture of 20% of the 96 h LC_50_ value with dried *Ocimum sanctum* leaf powder at 1.2 mg/L per day. These three sublethal concentrations, along with a control, were used for the 45-day chronic toxicity tests. In this study, all treatments were categorized into four different groups:

(i) Group 1. Fish without toxicant;
(ii) Group 2. Fish with toxicant at 10% of 96 h LC_50_ value;
(iii) Group 3. Fish with toxicant at 20% of 96 h LC_50_ value;
(iv) Group 4. Fish with toxicant at 20% of 96 h LC_50_ value and 1.2 mg/L leaf powder of *Ocimum sanctum*/day.

Each aquarium, after appropriate preparation, was stocked with ten acclimatized *Anabas testudineus*. Twenty-four hours after stocking, the fish were treated with the test chemicals. The stocked fish were fed with white fish meal, wheat flour, shrimp meal, dried yeast, and soybean meal, supplemented with vitamins and minerals, six days a week. Initially, the food ration was set at 2–3% of the fish’s stocking weight, with a 10% increase every two weeks. A separate experiment was conducted to observe changes in the reproduction of *Anabas testudineus*, with males and females stocked in a 1:1 ratio (mean body weight: 38.24 ± 5.56 g; mean total length: 12.3 ± 3.3 cm). The same management practices were followed during the rearing of male and female fish to monitor reproductive changes. Triplicates for all chronic toxicity tests were conducted in accordance with the established methods [7,8,30], with thirty fish exposed to each concentration of the test chemical.

For bioremediation, dried *Ocimum sanctum* leaf powder was administered at 1.2 mg/L per day with the fish food to mitigate the toxicity of the test chemical. During the toxicity tests, static renewal bioassay methods were employed, with 10% of the water in the test aquaria siphoned out every 24 h and replaced with fresh water containing the appropriate concentration of the test chemical. Results from the 45-day chronic toxicity tests were recorded on days 1, 15, 30, and 45. Alterations in biochemical parameters of blood serum (total glucose, protein, urea, creatinine, alanine aminotransferase-ALT, and aspartate aminotransferase-AST) and haematologicalparameters (RBC, WBC, Hb, and Hct %) were measured every 15 days. At the end of the experiment, changes in growth (including the gastrosomatic index, hepatosomatic index (HSI), weight gain, % increase in length, Specific Growth Rate, Food Conversion Ratio-FCR, and Food Conversion Efficiency-FCE), as well as reproductive parameters (gonadosomatic Index, ovary size, and fecundity), were evaluated. The physicochemical properties of test water (pH, CO_2_, dissolved oxygen, alkalinity, and hardness) were also assessed every 15 days during the chronic bioassay.

#### 2.4.2. Growth and Organo-Somatic Indices

The length and weight of the fish were noted within 24–48 h of preservation. The several growth parameters (gastrosomatic index, hepatosomatic index, percent increase in weight, percent increase in length, specific growth rate, food conversion ratio, food conversion efficiency) were evaluated from the length (mm) and weight (g) of the fish and their different tissues. The data of length and weight were transformed to log values for regression analysis. To calculate the growth parameters of the fish, the formulae were adopted from standard protocols [31,32,33] and they are given below:

(i) Gastrosomatic Index = (V/W) × 100,
  where V is the visceral weight (g) of the fish and W is the observed body weight (g) of fish;
(ii) Hepatosomatic index (HSI) = [{wet weight (g) of liver without gall bladder}/wet body weight (g)] × 100;
(iii) Percentage increase in weight = (W_2_ − W_1_)/W_1_ × 100,
  where W_1_ is the initial weight (g) of the fish and W_2_ is the final weight (g) of the fish;
(iv) Percentage increase in length = [(L_2_ − L_1_)/L_1_] × 100
  where, L_1_ = initial length of fish; L_2_ = final length of fish;
(v) Specific growth rate [34] (%/day) = {(loge W_2_ − loge W_1_)/T} × 100,
  where log_e_W_1_ = natural logarithm of initial body weight of fish (g), log_e_W_2_ = natural logarithm of final body weight (g) of fish and T = time interval;
(vi) Food conversion ratio (FCR) = food given/weight gain,
  where weight gain = final weight of fish (g) − initial weight of fish (g);
(vii) Gonadosomatic index of female fish = (G/W) × 100;
(viii) Fecundity = total number of ripening eggs/females.

#### 2.4.3. Haematological Biomarkers

The total count of RBC was performed following Dacie and Lewis [35]. For counting the red blood cells, whole blood from the test tube was pipetted by a hemocytometer tube up to the 0.5 mark and then diluted 200 times using Hayem’s fluid. After thoroughly mixing, the diluted blood sample was taken in the Neubaur’s counting chamber, covered with a special cover slip, and allowed to stand for a few minutes. The red cells were counted in the RBC counting chamber. For each sample, 5 readings were taken. The values were expressed in millions of cells/mm^3^. Hayem’s fluid was used as diluent for RBC, and Turk’s fluid was used for WBC. Hemoglobin was estimated using the acid haematin method [36]. Haematocrit (HCT), TWBCC, TRBCC, and Mean Corpuscular Haemoglobin [37] were expressed as follows:

- HCT (%) = (length of packed erythrocytes ÷ total length of blood column) × 100;
- TWBCC (103 mm^3^) = [total number of white blood cells counted in 4 squares of haemocytometer (Nwbc) × dilution factor (Df of 50)] ÷ [4 × volume factor (Vf of 0.1)];
- TRBCC (106 mm^3^) = [total number of RBCs counted in 5 squares of haemocytometer (Nrbc) × dilution factor (Df of 200)] ÷ [5 × volume factor (Vf of 0.1)].

#### 2.4.4. Biochemical Parameters of Blood Serum

Serum proteins were estimated following the methods of Lowry et al. [38]. The GOD/POD method (glucose oxidase-peroxidase) was employed for the estimation of glucose levels [39]. The enzymatic colorimetric method recommended by [40] was applied for the estimation of cholesterol. The creatinine level was estimated by following method [41]. The estimation of serum aspartate aminotransferase (AST) and serum alanine aminotransferase (ALT) was employed following the method of [42].

### 2.5. Scanning Electron Microscopic (SEM) Study

A heparinised syringe was used to take blood from the caudal peduncles of both control and polluted fish. Two to three blood drops were placed in vial with 2.5% glutaraldehyde produced in 0.1 M sodium cacodylate buffer. It was then centrifuged at 1500 rpm for 5 min, then washed and resuspended in distilled water. The procedure was performed 2–3 times. A thin film was decanted and applied to a cover slip after resuspension in distilled water. Then, the cover slips were air-dried and gold-coated using a JFC-1100 ion sputterer (JEOL Ltd., Tokyo, Japan). Observations were made on a JSM-6360 JEOL SEM at an accelerating voltage of 15–20 kV, using the secondary electron emission mode.

### 2.6. Homology Modelling and Molecular Docking

To predict the binding affinities of serum aspartate aminotransferase (AST) and alanine aminotransferase (ALT) with lead, molecular docking analysis was performed. The 3D structure of lead (PubChem CID: 5352425) was simulated using Avogadro software (version 1.2). Since the 3D structures of serum aspartate aminotransferase (AST; XP_026212619.1) and alanine aminotransferase (ALT; XP_026217554.1) were not available in the Protein Data Bank (PDB), we generated their structures through homology modeling using SWISS-MODEL [43]. A template search was conducted for these proteins, and models were built using ProMod3 [44]. The quality of the models was assessed using the QMEAN scoring algorithm at both the global and per-residue levels, and their validity was confirmed using Ramachandran plot analysis [45]. The final models were saved in PDB format.

For molecular docking, polar hydrogen atoms were incorporated into the target proteins to ensure proper ionization and tautomeric states of amino acids using the PyRx v8.0 tool [46]. Kollman united and Gasteiger charges were applied to the proteins, while the ligand was similarly prepared. The 3D structure of lead was further optimized in Open Babel, where its energy was minimized using the universal force field (UFF) with 200 steps of conjugate gradients. Universal and targeted docking simulations (50 runs each) were performed using PyRx [46] to identify potential binding sites, and the results were visualized using Discovery Studio v24.1.0.23298 [47].

### 2.7. Statistical Analysis

The Shapiro–Wilk test was performed to ascertain whether the data conformed to a normal distribution. All data, except those obtained from acute toxicity tests, were processed for significance of variance using one-way or two-way ANOVA (analysis of variance). Statistical analyses were performed using the SPSS package (version 16.0). The Probit Analysis [48] method has been employed for estimating LC_50_ values. The mean values of opercula movements obtained from respiratory tests were analysed by two-way analysis of variance (ANOVA), taking days of exposure and concentrations of a test chemical as independent variables. The comparison of mean values for significance of difference was performed using Dunnett’s test.

## 3. Results and Discussion

### 3.1. Acute Toxicity Assessment

#### 3.1.1. Effects on Mortality Rate

The results of acute toxicity test with Pb) to *Anabas testudineus* are shown in Table 1. The 24, 48, 72, and 96 h LC_50_ values of Pb were 1.47, 1.41, 1.15 and 1.08 mg/L, respectively. The 24, 48, 72, and 96 h LC_50_ values (with 95% confidence limits), probit regression equations and correlation coefficients (R^2^ and r values) of Pb indicate an existence of a strong positive correlation between the percentage of mortality and toxicant concentration (Table 1).

**Figure 1:**
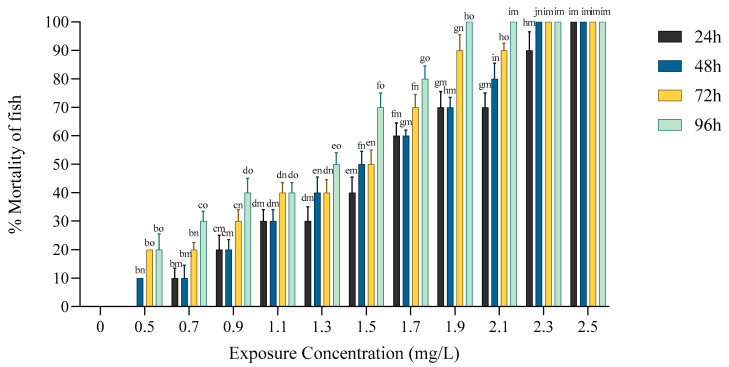
Mean percentage (%) values of mortality of *Anabas testudineus* exposed to different concentrations of lead over various exposure periods (24, 48, 72, and 96 h). Significant differences are denoted by b–i (columns) and m–o (rows).

No mortality was observed in the control group during 96 h experiment. The mortality of *Anabas testudineus* increased significantly (*p* < 0.05) with increasing toxicant concentrations and the progression of exposure time (Figure 1).

This observation indicated dose- and time-dependent mortality. The LC_50_ values decreased significantly with increasing exposure period of the toxicant.

In the present study, the 96 h LC_50_ value of lead was found to be 1.08 mg/L, which is lower than the 96 h LC_50_ value of other fish species, such as 44 mg/mL for *Oreochromis niloticus* [49] and 34.20 mg/L for *Labeo rohita* [50]. Therefore, this variations in lead toxicity to different fish species depends on species and the test condition. The results indicate that the tested fish *A. testudineus* was facing high lead toxicity.

#### 3.1.2. Behavioural Changes

The increasing mucous secretion and hyper-excitability were recorded at the higher concentrations of the test chemical (1.1 mg/L) from 72 h to 96 h of exposure time. The equilibrium of fish was lost at higher concentrations of the toxicant (1.1 mg/L and above) at 72 and 96 h of exposure (Table 2).

On initial exposure at higher concentration of lead, the fish showed characteristic avoidance behaviour by rapid and erratic swimming with jerky movements and hyper excitability, rapid opercula movement, jumping out from the test media, lateral swimming, and loss of equilibrium. These observations were in conformity with the findings of Murugan et al. [51], who observed characteristic restlessness, increased activity and opercular movements of fish put into hypoxic conditions. Similar abnormal behaviours were also observed in *Ctenopharyngodon idella*, *Clarias gariepinus*, and *Chanos chanos* exposed to lead nitrate [52,53].

#### 3.1.3. Changes in Respiratory Rate

**Figure 2:**
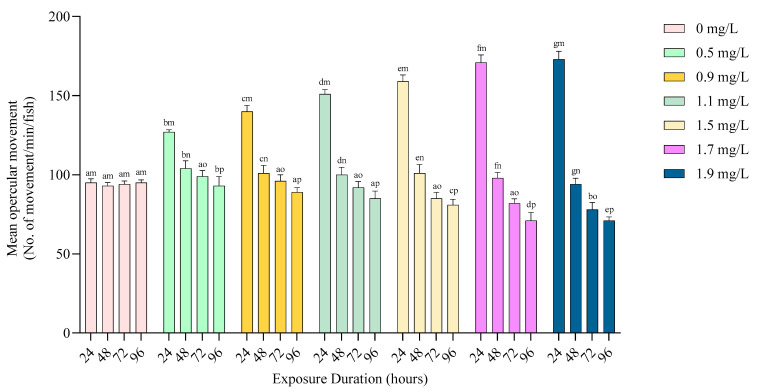
Mean opercular movements of *Anabas testudineus* during 96 h exposure to different exposure concentrations of Pb (mg/L). The significance levels are denoted using a–g (columns) and m–p (rows).

The opercular movement of fish increased significantly (*p* < 0.05) at 24 and 48 h but decreased significantly (*p* < 0.05) at 72 and 96 h with increasing toxicant concentrations. The opercular movement decreased significantly (*p* < 0.05) at all the treatments with the advancement of exposure time (Figure 2).

#### 3.1.4. Changes in Feeding Rate

**Figure 3:**
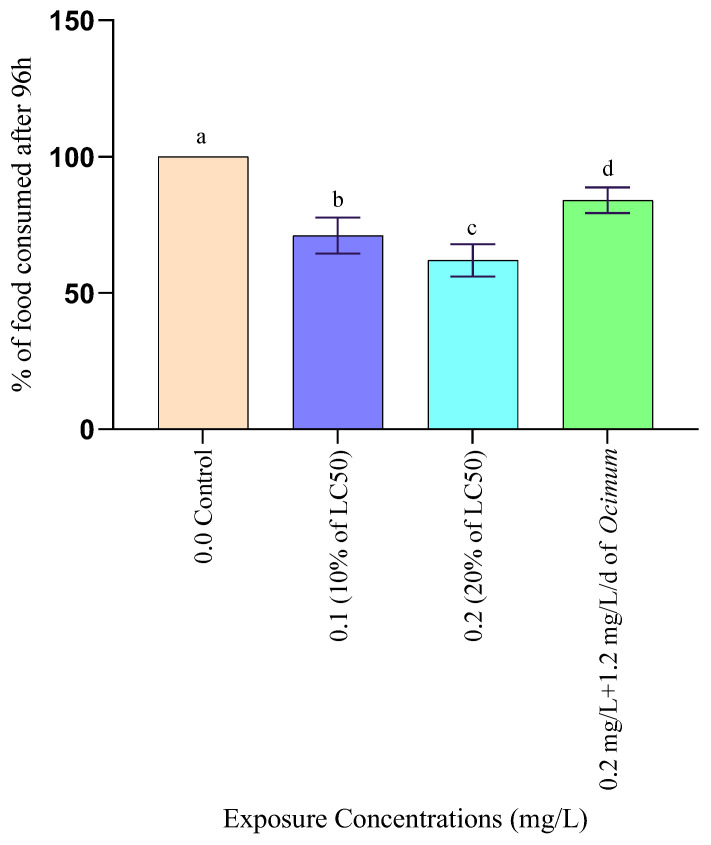
The proportion of food consumed by *Anabas testudineus* exposed for 96 h to different concentrations of Pb along with remediation with *Ocimum* leaf powder. Letters (a–d) indicate significant differences.

Changes in feeding rate of *Anabas testudineus* treated with different concentrations of Pb are shown in Figure 3. One-way ANOVA followed by Dunnett’s test showed that the feeding rate of fish was reduced significantly (*p* < 0.05) at 10% (0.1 mg/L), 20% (0.2 mg/L), and the mixture of 20% of the LC_50_ value of Pb and leaf powder of *Ocimum sanctum*. A severe decrease in feeding rate of fish was recorded at 20% of LC_50_ value of toxicant. But the rate of feeding of fish was significantly higher exposed to the mixture of 20% of LC_50_ value and leaf powder of *Ocimum sanctum* compared to the 10% and 20% LC_50_ concentrations of Pb (Figure 3).

### 3.2. Chronic Toxicity Tests of Lead to Fish Anabas testudineus

**Figure 4:**
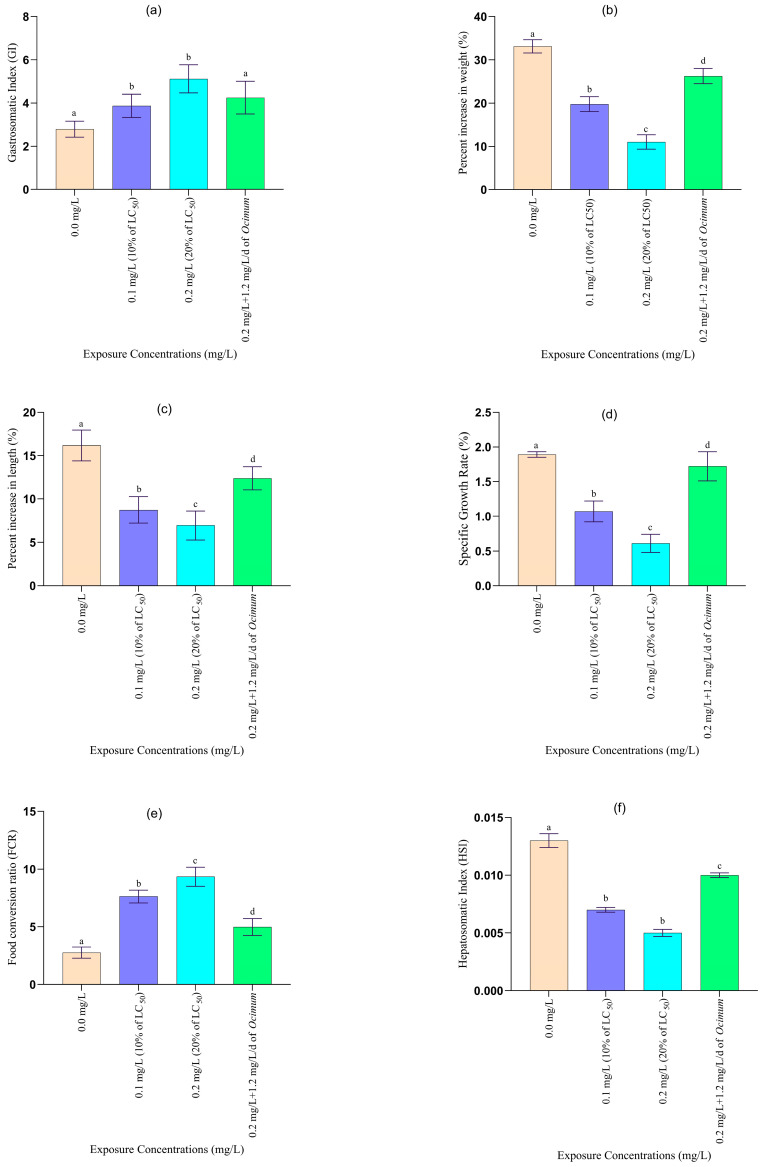

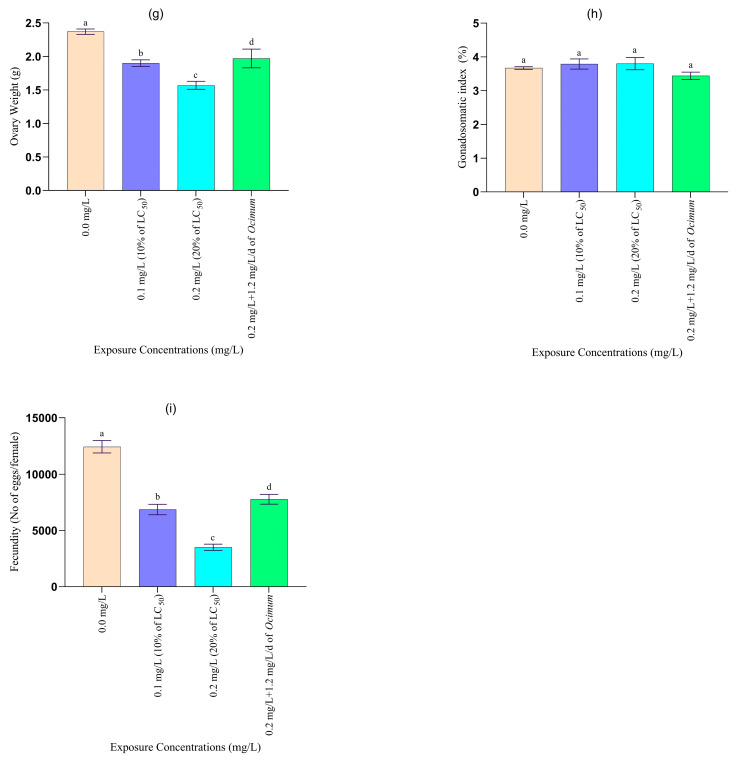
The (**a**) gastrosomatic index, (**b**) percentage increase in weight (**c**) percentage increase in length, (**d**) specific growth rate (% per day), (**e**) food conversion ratio (FCR), (**f**) hepatosomatic index, (**g**) gonad (ovary) weight, (**h**) gonadosomatic index (%), and (**i**) fecundity of *Anabas testudineus* exposed to sublethal concentrations of lead (mg/L) after 45 days of chronic toxicity testing are reported. Letters (a–d) indicate significant differences.

**Figure 5:**
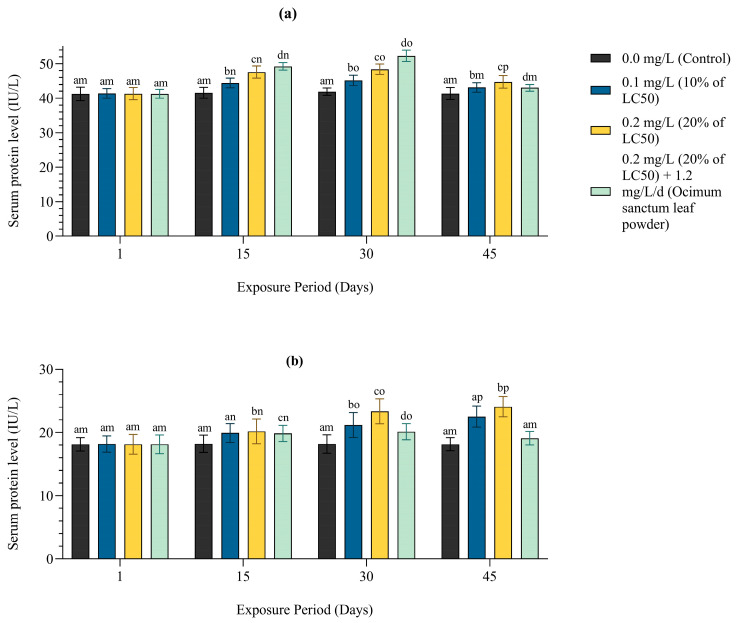
(**a**) Serum AST (IU/L) and (**b**) serum ALT (IU/L) level of freshwater fish *Anabas testudineus* exposed to Pb in the 45-day chronic toxicity bioassay. Letters a–g (columns) and m–p (rows) indicate significant differences.

No fish mortality was observed during the 45-day chronic toxicity bioassay. Additionally, no changes in behaviour or coloration were noted throughout the experiment. However, prolonged exposure (45 days) to sublethal concentrations of Pb resulted in significant alterations in growth rate, haematology, serum biochemistry, serum enzyme levels, and histopathology in the treated fish (Figure 4 and Figure 5 and Table 3 and Table 4).

#### 3.2.1. Changes in Growth and Organo-Somatic Indices

Long-term exposure (45 days) to sublethal concentrations of lead (Pb) resulted in significant alterations in various growth parameters of the treated fish compared to the control group (Figure 4). Chronic exposure to Pb at concentrations of 0.1 mg/L and 0.2 mg/L, as well as a mixture of 0.2 mg/L Pb and *Ocimum sanctum* leaf powder (1.2 mg/L/day), led to a significant decrease in food conversion efficiency, weight gain (%), specific growth rate, and the hepatosomatic index, along with a significant increase in the gastrosomatic index and food conversion ratio (FCR), compared to the control (*p* < 0.05).

The most severe reductions in food conversion efficiency, weight gain (%), specific growth rate, and hepatosomatic index were observed in fish exposed to 20% of the LC_50_ value (122 mg/L) of Pb. However, fish treated with the mixture of 20% LC_50_ Pb and *Ocimum sanctum* leaf powder exhibited a comparatively lesser reduction in these parameters than those treated with 10% or 20% Pb alone. Fish exposed to the combination of *Ocimum sanctum* leaf powder and 0.2 mg/L Pb showed increased growth in both length and weight compared to the groups treated with 10% and 20% LC_50_ Pb concentrations.

Moreover, fish treated with the mixture of *Ocimum sanctum* leaf powder (1.2 mg/L/day) and Pb (20% LC_50_ value) showed signs of recovery from Pb toxicity. Over the 45-day period, there was a gradual improvement in food conversion efficiency, weight gain (%), specific growth rate, and the hepatosomatic index, along with a gradual reduction in the gastrosomatic index and food conversion ratio (FCR), compared to fish treated with Pb alone.

The size of ovary, gonadosomatic index [54], and fecundity of *Anabas testudineus* were reduced significantly in the fish exposed to 10% and 20% of 96 h of LC_50_ values of lead in comparison to control. The rate of reduction in the above parameters was found directly proportional to toxicant concentrations. But no negative change in the above parameters was recorded in the mixture of 20% of 96 h of LC_50_ value of lead with 1.2 mg/L/d of leaf powder of *Ocimum sanctum* (Figure 4).

The chronic exposure of fish to 10% and 20% of LC_50_ of lead and a mixture of 20% of LC_50_ of Pb with *Ocimum sanctum* leaf powder alters the rate of weight gain (%), specific growth rate, and the hepatosomatic index. Fish performance and feed utilization, however, were significantly affected by lead concentration and exposure time. It also reported significant decrease in growth of *Channa punctatus* when exposed to heavy metal. Also, Abdel-Tawwab et al. [55] observed a significant decrease in the growth of Nile tilapia and common carp, respectively, when exposed to metal. Decreased growth in the post-larval stages of Indian prawn (*Penaeus indicus*) at 40, 80, and 160 µg/L was reported by Rajput et al. [56]. Dauble et al. [57] also observed reduced growth of fathead minnows exposed chronically to the toxicant. The hepatosomatic index is the main indicator of metabolic activity in animal organisms. In the present study, the recorded decrease of HSI values, due to lead exposure, indicates degenerative changes in the liver [58]. Bekmezciand Nevin [59] also reported that heavy metals decreased the hepatosomatic index in *Clarias gariepinus*, which was possibly due to depletion of energy reserves in liver. Stress condition developed under the effect of metals and the excess usage of energy reserves in response to increase in requirement might cause the decrease in hepatosomatic index [60].

Many heavy metals are considered essential nutrient elements that positively improve fish growth and feed utilisation; however, when these metals exceed the maximum allowable limit, they pose a risk not only to fish health but also to human consumers and disrupt ecological systems. Heavy metal toxicity has been linked to reduced gonadosomatic index [54], fecundity, hatching rate, fertilisation success, aberrant form of reproductive organs, and, ultimately, reproductive failure in fish. The rate of reduction in weight gain (%), specific growth rate, hepatosomatic index, gonadosomatic index [54], fecundity, and the rate of increase in the gastrosomatic index [54] and food conversion ratio (FCR) were the highest in fish treated with 20% of 96 h LC_50_ value of lead. The rate of change of these parameters was comparatively lower in the fish treated with a mixture of 20% of LC_50_ of lead and leaf powder of *Ocimum sanctum* over the fish treated with 10% and 20% of 96 h LC_50_ values of lead. The HSI, GSI, and fecundity were increased significantly in the mixture of 20% of 96 h of LC_50_ value of lead with 1.2 mg/L/d of leaf powder of *Ocimum sanctum* over 10% and 20% of 96 h of LC_50_ value of lead. It was probably due to the inhibitory effects of *Ocimum sanctum* reducing the oxidative stress and other toxicity from the lead during the chronic exposure of *A. testudineus* to the mixture of lead and *Ocimum sanctum* leaf powder. A similar result was also recorded by earlier researchers, such as Abdel-Tawwab et al. [61]. Similar findings were also recorded in HSI and GSI of *Heteropneustes fossilis* exposed to malathion and different metals [50]. A significant level of reduction in HSI of fish exposed to organic pollutants like PAHs was also found [62]. The HSI and GSI of *Cyprinus carpio* and *Perca fluviatilis* were also decreased during their exposure to lead and cadmium [63].

#### 3.2.2. Haematological Changes

Chronic exposure (45 days) to sublethal concentrations of Pb caused changes in different haematological parameters of treated fish as compared to control (Table 3). The results indicated that RBC, WBC, Hb, and Ht% decreased significantly (*p* < 0.05) in the fish treated with 0.1 and 0.2 mg/L of Pb and a mixture of 0.2 mg/L of Pb and *Oscimum sanctum* leaf powder (1.2 mg/L/d) compared to the control. The effects of Pb were severe in treated fish in comparison to control. The reduction of RBC, WBC, Hb and Ht% was comparatively lower in the fish treated in the mixture of 20% of LC_50_ of Pb and leaf powder of *Ocimum sanctum* over the fish treated with 10% and 20% of Pb. There was no significant change in Ht% in the fish treated with a mixture of 20% of LC_50_ of Pb and leaf powder of *Ocimum sanctum* compared to control. The fish treated with a mixture of *Ocimum sanctum* leaf powder (1.2 mg/L/d) along with Pb (20% LC_50_ value) showed a sign of recovery from the Pb toxicity with the gradual increase in RBC, WBC count, Hb, and Ht% over a period of 45 days compared to the treated fish.

In the present investigation, the RBC, WBC, Hb, and Hct (%) values of fish were altered during chronic exposure to lead. The reduction rate was dose- and time-dependent. The hematological parameter in fishes are frequently used for the assessment of the toxic effects as well as functional status of aquatic organisms by using blood, which is an excellent indicator of toxic stress [64]. The haematological parameters, including RBC, WBC, Hct value and Hb is generally used to assess the health status of fish [65,66]. In this findings, reduction in RBC, Hb and Ht content in fish upon lead exposure, may be because of the disorder in hematopoietic processes, accelerated disintegration of RBC cell membrane [67]. Normally increased WBC count in fish exposed to lethal and chronic doses indicates leucocytosis [68]. In this study, leukocyte count decreased as dose increases which are in accordance to findings of many researchers [69,70]. Moreover, use of *Ocimum sanctum* leaf powder along with lead abridged the harmful property of the toxicant in terms of haematologicalparameters. Similar findings were observed when treated fish were exposed to toxicant [71]. Therefore, it could be useful as a protective agent against lead induced toxicity in fish.

#### 3.2.3. Biochemical Changes of Blood Serum

Chronic exposure of fish to sublethal concentrations of Pb (0.1 mg/L, 0.2 mg/L and a mixture of 20% of LC_50_ of Pb and leaf powder of *Ocimum sanctum*) for 45 days caused noteworthy changes in different biochemical parameters (Table 4). The results indicated that serum aspartate aminotransferase (AST) and alanine aminotransferase (ALT) increased significantly (*p* < 0.05) in fish treated with 0.1 and 0.2 mg/L of Pb and a mixture of 0.2 mg/L of Pb and *Oscimum sanctum* leaf powder (1.2 mg/L/d) compared to control. The increase was dose and time dependent. There were also significant differences in serum enzyme activities between the two sublethal concentrations (0.1 and 0.2 mg/L) of Pb (*p* < 0.05). The fish treated with a mixture of *Ocimum sanctum* leaf powder (1.2 mg/L/d) along with Pb (20% LC_50_ value) showed a significant variation in the AST and ALT from 10% and 20% of LC_50_ values of Pb (*p* < 0.05), but no significant change was found compared to the control. This result may be considered as the sign of recovery from the Pb toxicity with the gradual decrease in serum ALT and AST over a period of 45 days compared to treated fish.

The utilisation of biochemical parameters in organisms as pollution indicators provides information on the adaptive or deleterious responses in organism exposed to a particular amount of chemicals. Such analyses provide early warning signals before other toxicological points, including death [72]. Among the biochemical profiles, plasma glucose has been widely used as a parameter to study stress and also used as a sensitive indicator of environmental stress in fish [73]. The hyperglycaemia recorded in the present study after lead exposure may be an indication of induced degenerative changes in the hepatopancreas [73]. The significant decrease in plasma protein levels in lead trioxide-treated fish might be due to an impaired rate of protein synthesis under metallic stress [74,75]. The variations in cholesterol level induced by heavy metals might be due to liver failure, which subsequently leads to the elevation of cholesterol concentration in the serum [76]. Various other metals also caused a decrease in the level of serum cholesterol in different fish species [77,78,79]. Serum creatinine is a traditional screening index for kidney function [55]. Similar results were obtained by [80], who found increased creatinine levels in Nile tilapia and common carp, respectively, due to metal toxicity.

#### 3.2.4. Change in Blood Serum Enzymes

A notable effect of Pb was noted in the different enzyme levels of *Anabas testudineus* after 45 days of exposure to different sublethal concentration of Pb and a mixture of Pb and leaf powder in comparison to the control (Figure 5). The results showed that serum aspartate aminotransferase (AST) and alanine aminotransferase (ALT) increased significantly (*p* < 0.05) in fish treated with 0.1 and 0.2 mg/L of Pb and a mixture of 0.2 mg/L of Pb and *Oscimum sanctum* leaf powder (1.2 mg/L/d) in compare to control. The increase was dose and time dependent. There were also significant differences in serum enzyme activities between the two sublethal concentrations (0.1 and 0.2 mg/L) of Pb (*p* < 0.05). The fish treated with a mixture of *Ocimum sanctum* leaf powder (1.2 mg/L/d) along with Pb (20% LC_50_ value) showed a significant variation in the AST and ALT from 10% and 20% of LC_50_ values of Pb (*p* < 0.05) but no significant change was found compared to the control. This result may be considered as the sign of recovery from the Pb toxicity with the gradual decrease in serum ALT and AST over a period of 45 days compared to the treated fish.

In the present study, ALT and AST levels in blood serum of the exposed fish increase with the increasing dose of lead and exposure time. Therefore, the effects of a chemical usually appear primarily in the liver [81]. Liver function tests have been used as indicators to access alterations in liver functioning following exposure to lead [81]. Several enzymes, such as ALP, GOT, and GPT, have been used to determine pollution exposure in animals and to monitor water pollution. In the present study, the significant increase in AST and ALT levels in fish exposed to lead indicates hepatic damage due to Pb accumulation, which, in turn, releases these enzymes into the bloodstream [81].

In the present study, the rate of elevation in ALT and AST levels was much higher in the fish treated with Pb and was comparatively lower in the fish treated with a mixture of 20% of LC_50_ of lead and leaf powder of *Ocimum sanctum* compared to the fish treated with 10% and 20% of lead. It indicates that the leaf powder of *Ocimum sanctum* decreases the Pb-induced toxicity of fish during their chronic exposure for a period of 45 days. In the present study, the use of leaf powder induced reversibility from abnormal levels to normal levels of ALT and AST in the blood of fish exposed to lead and may be regarded as a protective agent against toxicity. Similar findings were observed when the exposed animals were subjected to supplementation with *Moringa oleifera* extracts and spirulina [82,83].

#### 3.2.5. Scanning Electron Microscopic (SEM) Study on the Change in RBC of Fish

**Figure 6:**
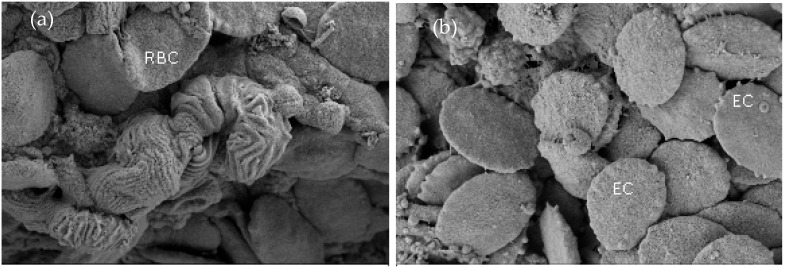
Scanning electron micrograph of RBC of *Anabas testudineus*: (**a**) RBC of control fish (**b**) RBC of fish treated with 0.2 mg/L (20% LC_50_ value) of lead.

Under scanning electron microscope, the erythrocytes of the treated fish showed an abnormal shape and became discoidal to elongated in shape with irregular surface (Figure 6). But the control blood cells were elliptical and discoid with normal smooth surface. Some erythrocytes of the treated fish formed lobopodian membrane protrusions. A few erythrocytes formed abnormal notches and spikes on the cell membrane.

The abnormally shaped RBCs of *Anabas testudineus*, exposed to lead with irregular surface, cell membrane crenation, internalization of membrane, oozed out cytoplasm, and lobopodian projections, were also found by earlier researchers in RBC of *Anabas testudineus* during chronic exposure to the pesticide cypermethrin [84]. Identical morphological changes in the RBCs of fish blood due to pesticides and heavy metal toxicity were also noted by several researchers [85,86]. The membrane internalization in mercuric chloride-exposed *Channa punctatus* was also observed [70]. The crenated RBC membrane development in fish might be due to the early echinocyte’s formation. The echinocytes development cause plasma membrane expansion leading to RBC swelling before lysing [87]. The crenation of lead-induced RBC membrane found in the present study may be associated with altered membrane surface area to cell volume ratio as stated by Naskar et al. [88] in *Clarias batrachus* exposed to aluminum. Similar types of crenations in the membrane of RBC were recorded in *Chana punctatus* exposed to mercuric chloride [70]. The formation of lobopodia and erythrocyte contraction from one side, as observed in the present study, has also been reported in *Channa punctatus* exposed to malathion and mercuric chloride [70]. Oozing cytoplasmic content and lobopodial projections were also found in the erythrocytes of *Anabas testudineus* exposed to the pesticide chlorpyrifos [84].

### 3.3. Homology Modelling and Docking Analysis of ALT and AST

For the modelling of ALT and AST, the templates obtained from the search have a template number of A0A3Q3AW13.1. ALT has a sequence identity of 87.37% and a GMQE score of 0.96 with 100% coverage, whereas AST has a sequence identity of 77.51% and a GMQE score of 0.92 with 100% coverage. A GMQE score above 0.7 is generally considered to be reliable [89].

**Figure 7:**
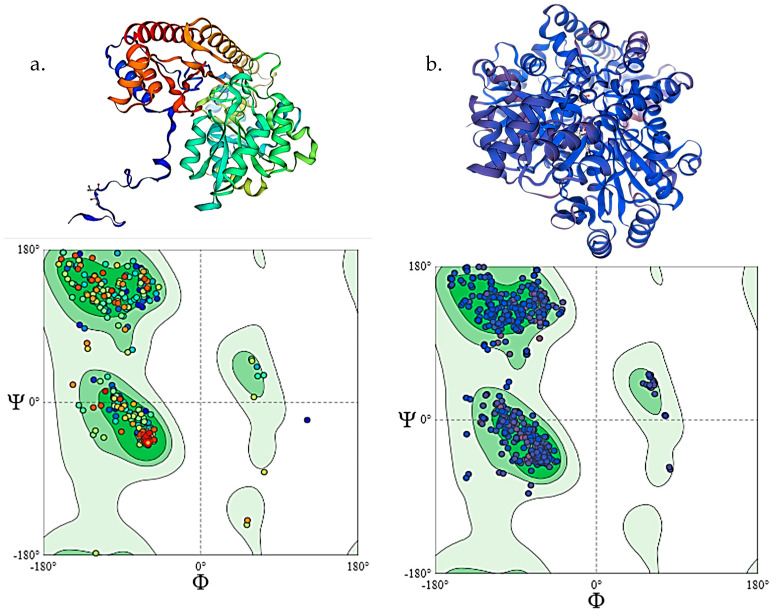
Homology models of ALT (**a**) and AST (**b**) with their validating Ramachandran plot below each of the models.

The structure assessment using the Ramachandran plot also shows the model’s validity. A valid protein is determined by 90–95% of its amino acids within the favoured regions of the plot, and here we obtained 97.96% amino acids in the favoured regions for ALT and 96.56% for AST. We obtained a MolProbity score of 0.65 for ALT and 1.65 for AST using MolProbity version 4.4 [90], and a clash score of 0.39 [44] for ALT and 1.39 for AST. The clash score was calculated as the number of collisions per 1000 atoms (including hydrogens) [91,92], and the MolProbity score signifies the combination of protein quality scores [44]. As the clash score and MolProbity score are low, the model can be considered suitable (Figure 7a,b). With these protein models (Figure 7a,b), we performed the docking analysis.

**Figure 8:**
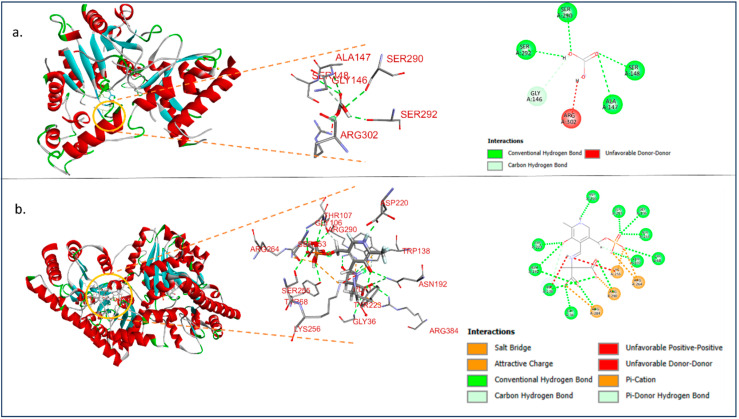
(**a**) Lead interacting with ALT and the 2D representation of the interaction at its left; (**b**) lead interacting with AST and the 2D representation of the interaction at its left.

The docking analysis of lead with ALT shows lower affinity to AST. In Table 5, all the predicted results are mentioned regarding binding affinities. Thus, AST might interact with lead much more than ALT (Figure 8a,b).

## 4. Conclusions

The present study aimed to assess the impact of lead exposure on *Anabas testudineus* in an aquatic ecosystem, with a focus on both its toxicity and the potential mitigating effects of *Ocimum sanctum*. In addition to evaluating lead’s harmful effects on *A. testudineus*, the study explored the role of *O. sanctum* in reducing this toxicity. Chronic toxicity tests revealed that lead concentrations of 0.1 mg/L (equivalent to 10% of the 96 h LC_50_) or higher reduced feeding rate, red blood cell count (RBC), hemoglobin (Hb), hematocrit (Hct) values, plasma glucose, and plasma protein levels, while decreasing white blood cell (WBC) count and serum ALT and AST levels. These physiological alterations, along with decreased appetite, were likely the primary factors contributing to the reduced growth of the fish during prolonged lead exposure. This suggests that *O. sanctum* may play a role in mitigating oxidative stress and other toxic effects of lead, indicating a potential recovery in the fish. These findings demonstrate the significant inhibitory effect of *O. sanctum* on lead toxicity. The results of this study provide critical data on both acute and chronic lead toxicity, which could inform national and international threshold levels for lead disposal in aquatic environments. Additionally, the findings offer valuable insights into sustainable fishery management, breeding programs, and the conservation of economically important native fish species, such as *A. testudineus*, in their natural habitats.

## Figures and Tables

**Table 1:** Comparison between the LC_50_ values (95% confidence limits, probit regression equation, R^2^ and r values) of Pb to *Anabas testudineus* at different times of exposure.

Time of Exposure	LC_50_ (mg/L)	95% Fiducial Limits of LC_50_		Probit Regression Equation Y = ax + b	R^2^ Value	r Value
		Lower	Upper			
24 h	1.47 ± 0.22	1.28	1.70	y = 4.41x + 4.25	0.9	0.9
48 h	1.41 ± 0.28	1.19	1.69	y = 3.54x + 4.45	0.9	0.9
72 h	1.15 ± 0.28	0.96	1.37	y = 3.53x + 4.78	0.9	0.9
96 h	1.08 ± 0.34	0.86	1.36	y = 2.91x + 4.90	0.8	0.8

**Table 2:** Impact of Pb on behaviours (MS: mucous secretion; HE: hyper-excitability; LE: loss of equilibrium) of *Anabas testudineus* at different hours (24, 47, 72, 96) of exposures; (-: absent; +: mild; ++: moderate; +++: strong).

Dose (mg/L)	MS				HE				LE			
	24 h	48 h	72 h	96 h	24 h	48 h	72 h	96 h	24 h	48 h	72 h	96 h
0	-	-	-	-	-	-	-	-	-	-	-	-
0.5	-	-	-	+	-	-	-	+	-	-	+	+
0.9	-	+	+	+	+	+	+	+	-	-	+	++
1.1	-	+	++	+++	+	+	++	+++	-	+	++	+++
1.5	-	+	+++	+++	+	+	+++	+++	-	+	+++	+++
1.9	+	++	+++	+++	+	++	+++	+++	+	+++	+++	+++

**Table 3:** Total RBC, total WBC, haemoglobin (g/dL) and Hct% of fish *Anabas testudineus* exposed to lead during chronic toxicity bioassay. Mean values with different superscript letters a–d within columns and m–p within rows are significantly different (two-way ANOVA followed by Dunnett’s test, *p* < 0.05).

Hematological Parameter	Exposure Time (d)	Concentration of F (mg/L)			
		0.0 mg/L (Control)	0.1 mg/L (10% of LC_50_)	0.2 mg/L (20% of LC_50_)	0.2 mg/L (20% of LC_50_) + 1.2 mg/L/d (*Ocimum sanctum* Leaf Powder)
Total RBC (10^6^/mm^3^)	1	2.83 ± 0.07 ^am^	2.84 ± 0.09 ^am^	2.84 ± 0.12 ^am^	2.83 ± 0.08 ^am^
	15	2.85 ± 0.16 ^am^	2.71 ± 0.07 ^bn^	2.65 ± 0.17 ^bo^	2.76 ± 0.2 ^bp^
	30	2.84 ± 0.16 ^am^	2.62 ± 0.16 ^bn^	2.53 ± 0.13 ^co^	2.78± 0.25 ^cp^
	45	2.83 ± 0.15 ^am^	2.51 ± 0.05 ^dn^	2.42 ± 0.16 ^do^	2.80 ± 0.15 ^dp^
Total WBC (10^3^/mm^3^)	1	9.72 ± 0.34 ^am^	9.71 ± 0.45 ^am^	9.72 ± 0.44 ^am^	9.73 ± 0.57 ^am^
	15	9.69 ± 0.43 ^am^	9.14 ± 0.34 ^bn^	9.01 ± 0.58 ^bo^	9.38± 0.37 ^bp^
	30	9.71 ± 0.22 ^am^	8.87 ± 0.43 ^cn^	8.53 ± 0.57 ^co^	8.59 ± 0.57 ^bp^
	45	9.72 ± 0.41 ^am^	8.58 ± 0.22 ^dn^	7.17 ± 0.82 ^do^	9.23 ± 0.48 ^cp^
Haemoglobin (g/dL)	1	14.92 ± 0.52 ^am^	14.91 ± 0.51 ^am^	14.91 ± 0.47 ^am^	14.92 ± 0.68 ^am^
	15	14.90 ± 0.43 ^am^	13.12 ± 0.29 ^bn^	12.02 ± 0.17 ^bo^	12.58 ± 0.47 ^bp^
	30	14.93 ± 0.24 ^am^	12.76 ± 0.58 ^cn^	11.38 ± 0.37 ^co^	13.44 ± 0.73 ^cp^
	45	14.92 ± 0.44 ^am^	11.69 ± 0.57 ^dn^	9.57 ± 0.36 ^co^	13.81 ± 0.27 ^dp^
Hct (%)	1	24.47 ± 1.35 ^am^	24.46 ± 0.96 ^am^	24.45 ± 1.35 ^am^	24.46 ± 0.99 ^am^
	15	24.44 ± 1.46 ^am^	21.52 ± 1.25 ^am^	20.37 ± 1.34 ^bn^	22.43 ± 1.38 ^bo^
	30	24.43 ± 1.57 ^am^	20.39 ± 0.94 ^bn^	19.53 ± 1.52 ^co^	22.87 ±1.96 ^bp^
	45	24.46 ± 1.39 ^am^	18.65 ± 0.93 ^cn^	17.21 ± 0.81 ^co^	23.15 ± 1.01 ^cm^

**Table 4:** Plasma glucose (mg/dL), plasma protein (mg/dL), cholesterol (mg/dL) and creatinine level (mg/dL) of fish *Anabas testudineus* exposed to lead during chronic toxicity test. Mean values with different superscript letters a–d within columns and m–p within rows are significantly different (two-way ANOVA followed by Dunnett’s test, *p* < 0.05).

Parameter	Exposure Time (days)	Concentration of Pb (mg/L)			
		0.0 mg/L (Control)	0.1 mg/L (10% of LC_50_)	0.2 mg/L (20% of LC_50_)	0.2 mg/L (20% of LC_50_) + 1.2 mg/L/d (*Ocimum sanctum* Leaf Powder)
Plasma glucose (mg/dL)	1	60.23 ± 0.97 ^am^	60.47 ± 0.74 ^am^	60.35 ± 1.03 ^am^	60.27 ± 1.02 ^am^
	15	61.14 ± 0.30 ^am^	62.32 ± 0.85 ^bn^	63.17 ± 0.68 ^bm^	62.15 ± 0.77 ^bo^
	30	61.03 ± 0.98 ^am^	65.83 ± 0.36 ^cn^	67.29 ± 0.77 ^co^	63.12 ± 1.26 ^cp^
	45	60.67 ± 0.69 ^am^	68.17 ± 0.51 ^dn^	70.18 ± 0.75 ^do^	61.74 ± 0.94 ^am^
Plasma protein (mg/dL)	1	3.21 ± 0.18 ^am^	3.24 ± 0.13 ^am^	3.23 ± 0.04 ^am^	3.22 ± 0.02 ^am^
	15	3.20 ± 0.16 ^am^	2.91 ± 0.06 ^bn^	2.62 ± 0.13 ^bo^	2.93 ± 0.15 ^bp^
	30	3.19 ± 0.16 ^am^	2.73 ± 0.11 ^cn^	2.43 ± 0.12 ^co^	2.95 ± 0.18 ^cp^
	45	3.20 ± 0.17 ^am^	2.62 ± 0.11 ^dn^	2.19 ± 0.11 ^do^	2.99 ± 0.17 ^dp^
Cholesterol (mg/dL)	1	192.25 ± 1.07 ^am^	191.78 ± 1.22 ^am^	192.63 ± 1.84 ^am^	192.12 ± 1.36 ^am^
	15	191.79 ± 1.09 ^am^	187.34± 1.43 ^bn^	182.13 ± 1.38 ^bo^	188.19 ± 0.96 ^bp^
	30	192.13 ± 1.27 ^am^	181.57 ± 1.36 ^cn^	176.70 ± 1.57 ^co^	183.22 ± 1.65 ^bm^
	45	192.37 ± 1.58 ^am^	177.72 ± 1.55 ^dn^	167.58 ± 1.26 ^do^	189.18 ± 1.25 ^cm^
Creatinine (mg/dL)	1	0.43 ± 0.049 ^am^	0.49 ± 0.074 ^am^	0.49 ± 0.046 ^am^	0.46 ± 0.063 ^am^
	15	0.49 ± 0.067 ^am^	0.69 ± 0.053 ^bn^	1.06 ± 0.055 ^bo^	0.88 ± 0.043 ^bp^
	30	0.46 ± 0.054 ^am^	0.88 ± 0.045 ^cn^	1.17 ± 0.064 ^co^	0.79 ± 0.062 ^cp^
	45	0.49 ± 0.036 ^am^	1.19 ± 0.036 ^dn^	1.28 ± 0.042 ^do^	0.53 ± 0.032 ^dm^

**Table 5:** The binding affinities of the proteins and chemicals similar to the dimethoate obtained from docking analysis using Vina Wizard.

Sl. No.	Proteins	Binding Affinities (kcal/mol)
1.	Serum alanine aminotransferase (ALT)	−4.0
2.	Serum aspartate aminotransferase (AST)	−3.5

## Data Availability

The datasets generated during the current study are available from the corresponding authors on reasonable request.
